# Notes on the Treatment of Syphilis

**Published:** 1875-08

**Authors:** E. N. Brush


ART. II.—*Notes on the Treatment of Syphilis.* By E. N. BRUSH, M. D.

1. Clinical Contributions to Syphilidology. By Drs. VAN BUREN and KEYES. (*Archives of Dermatology*, January, 1875.

2. On the Use of Mercury in the Late Stages of Syphilis. By F. R. STURGIS, M.D. *American Journal of the Medical Sciences*, January, 1875.

3. On the Antecedents and Treatment of Tertiary Syphilis. By C. R. DRYSDALE, M. D., London. (*The Doctor*, April 1, 1875.) (Pamphlet Baillière and Co.)

4. The Treatment of Syphilis. By CHRISTIAN BÆUMLER. *Art. Syphilis. Ziemssen's Cyclopædia.* Vol. III, p. 271.

5. On the Use of Iodide of Potassium in Syphilis. By JOSEPH R. BECK, M. D. *Philadelphia Medical Times* March 13, 1874.

6. On the Nature of Syphilitic Affections, and upon the Mercurial Treatment. (*Sur la nature des affections syphil. et sur le traitment mercuriel.*) (*Gaz. des Hôpitaux*, January 12, 1875.)

7. A New Anti-Syphilitic, Tay*u*ya. By M. STANISLAS MARTIN. (*Bull-Gén. de Thérap.*, No. 12, 1875.)

I. The two cases related in this article are briefly as follows:

1. A physician while a student, contracts a chancre. This is followed by a slight papular rash, for which beyond a laxative no medicine is taken. In time the rash disappeared and was not followed by a successor. Nine years afterwards a swelling appeared upon the cranium, this node remained hard and received no attention for *eleven years.* After a severe illness lasting several weeks, the node softened. It was opened but instead of healing the edges sloughed, leaving the bone bare over a circular area of more than two inches in diameter. At the time he was seen the size of the sequestrum could not be determined, as it had not yet separated.

2. A western gentleman contracted a urethral chancre, in one year he married and had one healthy child. In twelve years he had some ecthymatous spots upon his legs. He went to New York, and was treated by a specialist in veneral diseases by arsenic, but neither at this time nor previously did he take mercury. The sores on his legs got well and their site is occupied by scars character-ister of a syphilitic lesion. One year later at the age of forty-seven the patient had brain trouble, loss of memory, double vision, etc., accompanied by loss of flesh, and a poor condition of general health. His physician told him he must give up work and go to

Europe. This he did, taking the physician also. In Paris a prominent specialist in Nervous Diseases told him that he was threatened with apoplexy and agreed with his physician as to the impending softening. Time and Hygiene improved his condition and he returned to America. After some months he consulted Drs. Van Buren and Keyes, for "tertiary gummy infiltration of the soft palate with rapidly advancing destructive ulceration along the posterior border." Under Iodide of Potassium improvement commenced and progressed with great rapidity.

These two cases are such as will furnish plenty of material for reflection, and may be of some value to those who insist that the administration of mercury is the cause of the appearance or severity of the tertiary lesions.

The authors draw the following facts from these cases. That they go to strengthen the views that,

1. The evolution of syphilis may be mild and irregular, even where no mercury is used early in the disease.

2. With the mildest beginnings in syphilis (untreated,) the most terrible consequences may occur after years of quiescence.

3. The severity of tertiary lesions does not depend upon previous use of mercury.

4. The efficacy of iodide of potassium properly employed, is not dependent upon a previous use of mercury.

5. A father with syphilis may have a perfectly healthy child.

6. Specialists and experts are liable to be deceived as to the significance of symptoms in a given case, and their authoritative position should call for the exercise of extreme caution in the expression of opinion for their responsibility is great.

II. In this article Dr. Sturgis makes a plea for mercury in the treatment of syphilis. He does not wish to be considered as believing it to be a specific, but claims that it is the most trustworthy weapon which we have with which to fight the disease.

The point to which he calls attention, is the use of mercury in the later and deep lesions of syphilis, the so-called tertiary stage.

Two cases are reported in detail, in which the iodide of potassium was used for a long period with but little improvement, and in which a marked change for the better was noticed when mercury was administered.

Dr. Sturgis believes in large doses of iodide of potassium, continued until the result is reached or the patient's condition demand their suspension. His favorite method of administering the mercurial is by inunction, employing for this purpose the oleate of mercury of the twenty per cent. strength.

III. The paper by Dr. Drysdale is of great interest in connection with the use of mercury in syphilis. He has been for many years an opponent to the use of mercury in syphilis, but has become convinced by his own observations and the teachings of Fournier and Hutchinson, that mercury may be used with benefit. He still denies its value in the tertiary stage, but more extended observation may convince him of its value in these conditions. In view of the dogmatic and frequently intolerant assertions of the anti-mercurialists, statements such as these coming from a man holding the position that Dr. Drysdate does, are of especial interest.

IV. Over three hundred pages of Ziemssen's Cyclopædia are occupied with the discussion of syphilis from the pen of Prof. Bæumler. The author does not pretend to speak from his own authority, but aims rather to give a view of the more recent and generally accepted views upon this subject. As a whole, the section upon treatment is quite satisfactory, although the author seems, on page 108, to make a sufficiently clear distinction between the soft or non-infecting chancre, and the local sore of syphilis, his directions in regard to cauterization would lead the reader to suppose that he was of the opinion that thorough cauterization of the syphilitic sore would sometimes prevent the appearance of further symptoms. Those who follow this rule will find themselves doomed to disappointment, no amount of cauterization, we believe, will prevent the occurrence of syphilis after the contraction of a *true syphilitic chancre,* if the patient lives long enough. In those cases where this result does not follow, we hold that a mistake in diagnosis has been made, or that the case is one of those exceptionable ones in which the local sore is not followed by other appreciable symptoms for periods extending sometimes over twenty or thirty years.

Iodoform as a dressing for primary lesions and sores upon the genitals is mentioned. Its use is advised in phagednic ulcers, but Izard who has experimented considerably with this remedy, declares that while it is highly beneficial in certain other conditions in phagednic sores it is of no value.

The use of mercury is advised as soon as the syphilitic character is decided upon, the author believing with some that the secondary lesions may be greatly lessened or warded off altogether by such a course. The importance of the systematic conduct of the treatment is insisted upon. On page 290, the dose of van Swieten's liquor should read from one to two fluid *drachms* instead of *ounces*.

The various excellencies or defects of the different forms of mercury are dwelt upon to some extent, and their modes of administration detailed.

Iodine and its various preparations and the treatment of the late stages of syphilis receive due attention. Nothing new is said in reference to the treatment of hereditary syphilis. Of syphilization he says: "The method is not likely to obtain the confidence of physicians or meet with the approbation of patients."

V. It is a pity that Dr. Beck did not read the two cases reported by Drs. Van Buren and Keyes (I) before writing his article and attempting to furnish food for contemplation. The two cases that he cites as proving the lack of value of mercury and the superior importance of the treatment by iodide of potassium are clearly testiary in character, and do not present anything astounding in the fact that they *improved* (Dr. Beck says were entirely *cured*,) under the use of iodide of potassium alone.

VI. The paper of Herman is a curiosity which aptly illustrates the extreme views held by some anti-mercurialists. He draws three conclusions:

1. Syphilis is a local disease, the consecutive forms of which bear close relations to the original lesion.

2. Mercury should be banished from its treatment.

3. What are termed constitutional lesions are the effects of mercury.

VII. Tayuya, the new anti-syphilitic announced by M. Martin, is of South American origin, and the stories told of it resemble those told of cundurango, and the new aphrodisiac damiana. We are not inclined to condemn it, however, without trial, and shall watch for further communications from M. Martin. He says that M. Ubicini, while traveling through Brazil, came across a population of negroes who used this remedy in the cure of syphilis. M. Martin publishes the results of some eight experiments made by him to discover the origin of the bitter taste of the plant. No alkaloid was discovered; however, besides a green resin brown extractive matter, tannin glucose starch and oil, the mineral substances, alumina, iron, lime, potash, etc., are so numerous that they fall in a white powder when an aqueous decoction is acidulated with acetic acid. M. Martin promises further details in a future number.

